# Chelation-Assisted Substrate-Controlled Asymmetric Lithiation-Allylboration of Chiral Carbamate 1,2,4-Butanetriol Acetonide

**DOI:** 10.3390/molecules20069890

**Published:** 2015-05-28

**Authors:** Adeem Mahmood, Hamad Z. Alkhathlan, Saima Parvez, Merajuddin Khan, Sohail A. Shahzad

**Affiliations:** 1 Department of Chemistry, College of Science, King Saud University, Riyadh 11451, Saudi Arabia; E-Mail: mdk.chem@gmail.com; 2 Department of Chemistry, COMSATS Institute of Information Technology, Abbottabad 22060, Pakistan; E-Mail: sohail_chem@yahoo.com; 3 School of Medicine, Shandong University, Jinan 250012, China; E-Mail: daring_saima@hotmail.com

**Keywords:** lithiation, borylation, allylation, chelation

## Abstract

The lithiation of 2-(2,2-dimethyl-1,3-dioxolan-4-yl)ethyl diisopropylcarbamate (**1**) is achieved freely by *sec*-butyllithium in diethylether with high *lk*-diastereoselectivity: the bicyclic chelate complexes **3a** and **3b** are reacted with electrophiles to form optically active precursors **4a** and **4b** with >95% diastereoselectivity. In addition, tertiary diamines can undergo an external complexation in contest with the internal oxygen ligand, leading to improved stereoselectivities. The further reactions of lithiated carbamates with trans alkenyl-9-BBN derivatives after 1,2 metallate rearrangements, gave the key intermediate α-substituted allylic boranes **7**. Subsequent allylboration of aldehydes gave (*Z*)-*anti-*homoallylic alcohols **8** in good yield and excellent *d.r*.

## 1. Introduction

Hoppe and co-workers investigated the lithiation of carbamates derived from non-racemic chiral primary alcohols generating organolithium intermediates which undergo electrophile-dependent stereodivergent substitution that often have remarkable configurational stability [1,2,3,4]. This stability is due to dipole stabilization and intramolecular chelation of the lithium counterion by the carbamoyl group [5]. The importance of carbamate group, in the enhancement of the kinetic acidity of α-protons and the stabilization of lithio derivatives by chelation through one of the oxygen lone-pairs has been recognized by many research groups [1]. It has been shown that the sterically congested 1,2,4-butanetriol acetonide carbamate could be lithiated by *s*-BuLi/TMEDA in diethylether to form an α-lithiated species [1,2], whilst in the presence of the chiral diamine (−)-sparteine, *pro*-*S* H deprotonates preferably and the configurationally stable lithiated complexes are subsequently trapped with different electrophiles with retention of configuration [6]. Generally, it has been considered that the remote donor substituents or groups, such as acetonide group, could also interfere in lithiation [7,8].

Aggarwal and co-workers have investigated the chelation-assisted substrate-controlled (CASC), asymmetric lithiation, and allylboration through less-sterically demanding chiral carbamates and explored its application for selectively making C-C bonds [9,10,11,12,13,14,15,16,17,18]. Previously, we have described this methodology to obtain highly selective products through substituted boranes (*trans*-alkenyl-9-BBN) and boronic esters and their reactions with sparteine-complexed lithiated carbamates [19]. Moreover, this protocol was further applied in the formation of penta-substituted tetrahydropyrans through the Prins cyclization [20].

## 2. Results and Discussion

In this paper, we optimized the use of the important enantioenriched stereodirecting carbamate (*S*)-2-(2,2-dimethyl-1,3-dioxolan-4-yl)ethyl diisopropylcarbamate (**1**) in lithiation, electrophilic addition, borylation and allylation reactions, involving two different protocols e.g., with/without the addition of an external bi-dentate diamine, such as TMEDA/(−)-sparteine/(+)-sparteine surrogate. This carbamate **1** is only differs in the leaving group moiety *i.e*., an open chain OCb instead of the cyclic OCby, from Hoppe’s carbamate used nearly 20 years back [3].

### 2.1. Chemistry

**Scheme 1:**
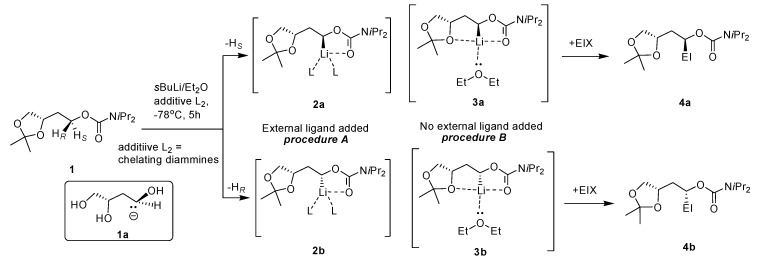
Chelation-directed lithiation/ES of carbamate **1**.

Two procedures were adopted for the deprotonation of the carbamate **1**, one in the presence of external ligands (*procedure A*) and another with no external ligands involved (*procedure B*) (Scheme 1). Although very similar results were obtained as per Hoppe [3], but products more bench stable (no isomerization occured even at longer time and elevated temperatures), and reactions are quicker, and give high yields and good *d.r*. This shows that the expulsion of an unhindered carbamate leaving group (OCb) could be quicker than that of a bulky carbamate (OCby). The protocol involved the Chelation-Directed-Asymmetric-Lithiation (CDAL) of 1,2,4-butanetriol acetonide by the drop-wise addition of *s*BuLi in Et_2_O or *s*BuLi in Et_2_O/additative chelating ligands e.g., *N*,*N*,*N*,*N*-tetramethylethylenediamine (TMEDA), (−)-sparteine or (+)-sparteine surrogate. An effective substrate-inherent chiral induction was exploited with acetonide-type carbamate **1**. Here we report on the generation of chiral synthetic equivalents for the 1,2,4-trihydroxybutanide ion **1a**, showing the stereo-directing influence of the protected 3-hydroxy group. Furthermore the conformational strain of the dioxalane ring is noteworthy for attaining good diastereoselectivities.

The relative rate for the deprotonation of the diastereotopic protons (*pro*-*S* or *pro*-*R*) reflects the rate-ratio in the presence of chiral inductor (diamine additives). The configurationally stable chiral ion pairs or lithio-intermediate **2a** originates preferably when we used (−)-sparteine as an external chiral bi-dentate ligand, following the abstraction of the *pro*-*S* proton. Subsequent trapping of this configured complex **2a** with different electrophiles (with retention of configuration) would then furnish **4a** in good yield and high *d.r*. (Table 1).

**Table 1:** The chelation-directed asymmetric lithiation of carbamate **1**. 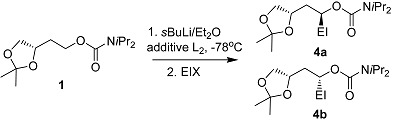

Entry	EIX	Additive L_2_	Yield ^a^ (%)	*d.r.* ^b^ 4a:4b	${\left[\text{α}\right]}_{D}^{23}$ (conc., CH_3_OH)
I	PhCHO	TMEDA	69	51:49	nd ^c^
		(−)-Sp	74	98:2	−17.4 (1.0)
		(+)-Sp surrogate	53	20:80	nd
		None	84	98:2	−17.2 (1.0)
II	Me_3_SnCl	TMEDA	80	53:43	nd
		(−)-Sp	75	97:3	+13.0 (1.2)
		(+)-Sp surrogate	nd	nd	nd
		None	78	98:2	+11.3 (1.1)
III	PhCOPh	TMEDA	72	50:50	nd
		(−)-Sp	80	95:5	+23.6 (1.0)
		(+)-Sp surrogate	61	29:71	nd
		None	84	95:5	+27.3 (1.5)

^a^: Isolated after chromatographic purification; ^b^: Relative stereochemistry determined from the crude product ^1^H-NMR spectra; ^c^: Not determined.

Indeed, the opposite diastereomer **4b** to (−)-sparteine can be effectively achieved through the appropriate choice of chiral diamines employed ((−)-sparteine, or (+)-sparteine surrogate) [21,22,23] (*procedure A*), whilst the racemic mixture with 1:1 ratio of the diastereomeric complexes **2a** and **2b** arises with *sec*-butyllithium/TMEDA. Interestingly, the diastereomeric ratio in **4a** and **4b** adequately increased to 98:2 when the lithiation was done without the addition of any external chelating diamine (*procedure B*).

From these results, it is clearly indicated that the intra- and intermolecular complexation involved an almost similar rate in the kinetically controlled deprotonation of the diastereotopic H*_S_* and H*_R_* protons in the carbamate ester. If no external bidentate ligands are linked to it then the β-oxygen atom acts mainly as a ligand to the lithium cation providing the bicyclic chelate complexes **3a** or **3b**, where two neighboring five-membered rings are *trans*-annulated to the central ring, in contrast to the intermediates **2a** or **2b** which are stabilized through intermolecular complexation with an external bidenate ligand e.g., TMEDA or (−)-sparteine or (+)-sparteine surrogate. It is noteworthy that chiral induction [24] arises in the deprotonation step due to the presence of a good donor substituent next to a stereogenic C atom in the γ*-* or δ-position and therefore high substrate-controlled diastereoselectivities can be easily achieved. A bicyclic chelate complex of the type **3a** is generated in the presence of (−)-sparteine, even if the specific stereochemistry of the bis-chelate complexes like **2a**, **2b** or **3a**, **3b** is doubtful. It is likely that a seven- or eight-membered ring might form due to the fixing of the more effective coordinating carbonyl group of the γ- or δ-carbamoyloxy residue. In addition, Et_2_O coordinates in monodentate fashion and hence the more favorable *exo*-position of the electrophile determines the transition state. It is believed that the formation of the *anti*-annulated tricyclic chelate complex **3a** is highly selective despite the fact that the lithiation step is kinetically controlled, whilst, in the presence of external ligands e.g., (−)-sparteine, due to the mismatched pair situation; the connection towards the abstraction of the *pro-S* proton is further enhanced, because in this case **3b** is no longer evident in the reaction mixture. The 2,2-dimethyl-substituted 1,3-dioxolane ring in the carbamate **1** is simply a weak ligand for lithiation, and as with sparteine, it is expelled by TMEDA. As Table 1 shows, diastereoselectivity is reduced under these conditions and is reversed by means of (+)-sparteine surrogate due to preference for the *pro*-*R* proton [25]. Furthermore the normal deprotonation pathway relates to the presence of a bidentate complexing ligand where intramolecular complexation is not concerned. Also the ion pair **3a** reacts efficiently with electrophiles and consequently the substrate generates a valuable synthetic equivalent to the (*S*)-1,3,4-trihydroxybutanide **1a** [26,27].

The second part of this paper illustrates the allylboration of aldehydes to form C-C bonds with control over relative and absolute stereochemistry (Table 2) [28,29,30,31,32,33,34,35,36,37,38,39,40]. Thus a wide scope and convergent method has been introduced for reacting lithiated carbamates with vinylboranes/boronic esters and aldehydes to give 1,2,4-trisubstituted homoallylic alcohols with full stereo control [19]. It has been described that by applying α-substituted allyl boranes, it is possible to control the initial double bond geometry and all three element of stereochemistry of the homoallylic alcohol products (enantioselectivity, *E*/*Z*-stereochemistry and *syn*/*anti* stereochemistry) without any additional stereodirecting reagents.

The present strategy involves the reaction of lithiated carbamate **2** with alkyl substituted *B*-vinyl-(9-BBN) **5** to prepare potentially the key intermediate α-substituted allylborane **7** through 1,2-metallate rearrangement product **6**. The subsequent allylation reaction with different choices of aldehydes (e.g., PhCHO, CyhexCHO and *n*BuCHO) gave exclusively the *anti-*(*Z*)-homoallylicsec-alcohols **8**. No isomerization of the labile α-substituted allylic borane **7** during the 1,2-metalate rearrangement was observed. This could be achieved by simply adding the aldehyde to the ate-complex **6** at low temperature after 1,2-metalate and rearrangement would occur upon warming to give the allyl borane **7** which would subsequently undergo allylation to give **8** before isomerization could occur. This protocol was found to be successful with a range of representative *trans-*vinylboranes **5a** and **5b**, carbamate **1**, and aldehydes (Table 2). In all cases, good diastereomeric ratios with moderate yields were observed.

**Table 2:** Synthesis of *anti*-(*Z*)-homoallylic alcohols through the lithiation/allylboration method. 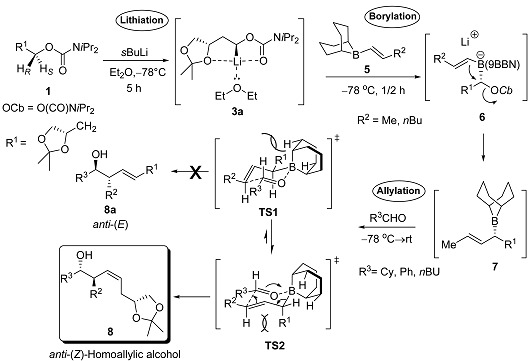

Entry	R^2^	R^3^	Yield (%)	*d.r*. ^a^ 8:8a	${\left[\text{α}\right]}_{D}^{23}$ (conc., CH_3_OH)
I	Me	Ph	56	90:10	+10.4
II	Me	Cy	54	95:5	−19.5
III	Me	Bu	60	92:8	+21.9
IV	Bu	Ph	48	90:10	−14.5
V	Bu	Cy	52	94:6	−18.4

^a^: Relative Stereochemistry determined by the crude product ^1^H-NMR-spectra.

The high selectivity originates from the closed 6-membered chair transition-state structures involved in minimizing non-bonded steric interactions [19,41], which can be rationalized by the increased steric hindrance in the transition-state structure **TS2** compared to **TS1** [42]. Severe steric hindrance between the 9-BBN ring and R^1^ would push the α-carbon substituent into a pseudo-axial position resulting in the *anti-*diastereoisomer and (*Z*)-alkene geometry [43,44,45,46]. It is interesting to note that external complexation of tertiary diamines can compete with the internal oxygen ligand, furnishing stereoselectivities with good diastereoselectivities in the desired compounds.

## 3. Experimental Section

### 3.1. General Information

All air- and water-sensitive reactions were carried out in oven-dried (180 °C) glassware and under an Air atmosphere using standard Schlenk techniques. Anhydrous solvents were prepared using a Grubbs-type anhydrous solvent drying columns.^1^H- and ^13^C-Nuclear Magnetic Resonance (NMR) spectra were acquired at various field strengths as indicated, and were referenced to CHCl_3_ (7.27 and 77.0 ppm for ^1^H and ^13^C, respectively) or TMS (0.00 ppm for ^1^H and ^13^C). ^1^H- and ^13^C-NMRspectra are shown in the Supplementary Materials. ^11^B-NMR spectra were recorded with complete proton decoupling using BF_3_·Et_2_O (0.00 ppm) as an external standard. Assignment of signals in ^1^H- and ^13^C-spectra was performed using ^1^H-^1^H COSY, DEPT, HMQC and HMBC, where appropriate. Low- and high-resolution mass spectra were recorded using Electron Impact (EI), Chemical Ionization (CI) or Electron-Spray Ionization (ESI) techniques. For CI, methane or NH_4_OAc/MeOH were used. Analytical TLC: aluminum backed plates pre-coated (0.25 mm) with Silica Gel 60 F_254_ (Merck Millipore, Darmstadt, Germany). Compounds were visualized by exposure to UV-light or by staining with 5% solution of (NH_4_)_2_Mo_7_O_24_**^.^**4H_2_O in EtOH followed by heating. Flash chromatography was carried out using Merck silica Gel 60, 0.040–0.063 mm particle size. Melting points were determined with a Boetius hot stage apparatus and were not corrected. All IR data were obtained on a Perkin-Elmer Spectrum One FT-IR spectrometer (Perkin-Elmer, Boston, MA, USA). *N*,*N*,*N*,*N*-Tetramethylethylenediamine (TMEDA) was purchased from Sigma-Aldrich (Gillingham, UK) and (−)-sparteine was purchased from Aldrich (5 years back from Gillingham, UK). (+)-sparteine surrogate was synthesized from commercially available (−)-cytisine [21]. Both were distilled under reduced pressure over CaH_2_ prior to use. 9-((*E*)-Prop-1-enyl)-9-borabicyclo[3.3.1]nonane (9-BBN) as dimer was purchased from Aldrich. *s*BuLi (1.3 M solution in cyclohexane/hexanes, 92:8) was purchased from Sigma-Aldrich.

### 3.2. (S)-2-(2,2-Dimethyl-1,3-dioxolan-4-yl)ethyl Diisopropylcarbamate *(**1**)*

To a solution of *N*,*N*-diisopropylcarbamyl chloride (8.80 g, 55.0 mmol) and NEt_3_ (8.15 mL, 58.0 mmol) in CH_2_Cl_2_ (100 mL) was added (*S*)-3-acetonide-ethyl-1-ol (7.60 mL, 55.0 mmol). This mixture was then heated to reflux and stirred for 24h. The reaction was cooled to rt. and H_2_O (100 mL) was added. The aqueous layer was extracted with CH_2_Cl_2_ (3 × 75 mL) and the combined organic layers were dried (MgSO_4_) and concentrated *in vacuo* to give the crude product as an orange oil. The crude oil was purified by flash chromatography (10% EtOAc/petroleum ether) to yield **1** as a colourless oil (6.64 g, 54%). *R*_f_ = 0.34 (15% EtOAc in petroleum ether); ${\left[\text{α}\right]}_{D}^{24}$ –24.3 (*c* 0.7, CH_2_Cl_2_); IRν_max_ (neat)/cm^−1^ 2972, 1690, 1442, 1312, 1218, 1069; ^1^H-NMR (CDCl_3_, 270 MHz); δ: 4.21 (2 H, d, *J* = 5.7 Hz, C*H*_2_OC), 4.08 (2 H, t, *J* = 6.1 Hz, CH_2_C*H*_2_O), 3.98–3.61 (2 H, br. m, 2 × C*H*), 3.58–3.52 (1 H, m, CH_2_C*H*O), 1.92–1.89 (2 H, m, CHC*H*_2_CH_2_), 1.37 (3 H, s, C*H*_3_), 1.31 (3 H, s, C*H*_3_), 2.00 (12 H, d, *J* = 6.8 Hz, 4 × C*H*_3_); ^13^C-NMR (CDCl_3_, 69.5 MHz); δ: 157.6 (*C*=O), 109.5 (O*C*(CH_3_)_2_), 74.8 (CH_2_*C*HO), 67.7 (CH*C*H_2_O), 57.0 (*C*H_2_O), 54.8 (2 × *C*H), 31.5 (*C*H_2_), 26.8 (2 × *C*H_3_), 21.2 (4 × CH_3_); HRMS(ESI) calcd. For C_14_H_27_NO_4_Na: 274.2011; Found: 274.2018; Anal. Calcd for C_14_H_27_NO_4_: C, 61.51; H, 9.96; N, 5.12; Found: C, 61.48; H, 9.98; N, 5.09.

### 3.3. 9-((E)-Prop-1-enyl)-9-borabicyclo[3.3.1]nonane *(**5a**)*

Following the method of Soderquist [47], propyne (6–10 mL) was condensed in a Schlenk flask, to which was added 9-BBN (0.5 M in THF, 3.76 g, 60.0 mL, 30.0 mmol). After addition the mixture was stirred at 0 °C until the white solid disappeared. Two hours later, the excess propyne and solvent were removed under reduced pressure, and the residue was subjected to high vacuum (0.1 mbar) through a dry ice-acetone condenser. The low boiling point component (the literature reported boiling point of was 66 °C/0.9 Torr) [47] was collected as a colourless oil (2.95 g, 56%). All spectral data matched those reported in the literature [45]. ^1^H-NMR (300 MHz, CDCl_3_) δ: 6.80 (1H, dq, *J =* 17.2, 6.4 Hz, HC=C*H*CH_3_), 6.23 (1H,dq, *J =* 17.2, 1.5 Hz, BC*H*=), 1.94 (3H, dd, *J =*6.4,1.5 Hz, C*H*_3_), 1.90–1.79 (6H, br. m, 3 × C*H*_2_),1.72–1.60 (6H, m, 3 × C*H*_2_),1.28–1.17 (2 H, m, 2 × C*H*); ^13^C-NMR (75 MHz, CDCl_3_); δ: 151.0 (2 × CH=CH), 33.8 (2 × CH), 24.6 (2 × CH_2_), 23.6 (4 × CH_2_), 22.1 (*C*H_3_); HRMS (CI) calcd. for C_11_H_20_^11^B: 163.1580; Found: 163.1582; ^11^B-NMR (96 MHz, CDCl3) 77.

### 3.4. B-(trans-1-Hexenyl)-9-borabicyclo(3,3,1)nonane *(**5b**)*

Following the method of Brown [48], 9-BBN (0.5 M in THF, 40.0 mL, 20.0 mmol) was added drop-wise to pre-degassed 1-hexyne (3.60 g, 5.0 mL, 44.0 mmol) at 0 °C. After addition, the reaction mixture was warmed to room temperature and stirred for two hours. The solvent was removed under reduced pressure and the crude product was purified by distillation under reduced pressure to give the borane as a pale yellow oil (2.58 g, 60%). B.p. 83–90 °C/0.15 mbar (lit [48] B.p. 72–74 °C/0.03 mbar). All spectral data matched those reported in the literature [45,48]. ^1^H-NMR (400 MHz, CDCl_3_); δ: 6.83 (1H, dt, *J* = 17.2, 6.4 Hz, HC=C*H*CH_2_), 6.24 (1H, dt, *J* = 17.2, 1.5 Hz, BC*H*=CH), 2.28 (2H, dt, *J* = 7.0, 6.4 Hz, C*H*_2_CH=CH),1.91–1.84 (6H, m, 3 × C*H*_2_), 1.77–1.67 (6H, m, 3 × C*H*_2_), 1.51–1.43 (2H, m, 2 × C*H*),1.41–1.33 (2H, m, C*H*_2_), 1.31–1.22 (2H, m, C*H*_2_CH_3_), 0.92 (3H, t, *J* = 7.2 Hz, C*H*_3_); ^13^C-NMR (75 MHz, CDCl_3_); δ: 156.3 (2 × *C*H=CH), 35.9 (2 × *C*H), 33.8 (2 × *C*H_2_), 33.7 (4 × *C*H_2_) 30.7 (*C*H_2_), 23.6 (*C*H_2_), 22.5 (*C*H_2_), 14.1 (*C*H_3_); HRMS (CI) calcd. for C_14_H_26_^11^B: 205.2049; Found: 205.2050; ^11^B-NMR (96 MHz, CDCl_3_) 77.

### 3.5. (R)-3-((S)-2,2-Dimethyl-1,3-dioxolan-4-yl)-1-hydroxy-1-phenylpropan-2-yl Diisopropylcarbamate *(**4a-I**)*

To a solution of carbamate(0.208 mg, 0.75 mmol) in Et_2_O (5 mL) at –78 °C was added *s*BuLi (1.3 M in cyclohexane, 0.84 mL, 1.05 mmol,) drop-wise. This mixture was then stirred at –78 °C for 5 h followed by addition of benzaldehyde (2.00 mL, 2.00 mmol). The reaction was stirred for 2h and then warmed to r.t. and stirred for 12h.The reaction mixture was then cooled to 0 °C and a solution of 2 N HCl (10 mL) was added drop-wise. The layers were separated and the aqueous layer was extracted with Et_2_O (3 × 15 mL). The combined organic layers were dried over MgSO_4_ and concentrated *in vacuo*. The crude product with four pairs of diastereomers was purified by flash chromatography (SiO_2_, 10% EtOAc/petroleum ether) to give the major diastrereomeric product **4a**-**I** (196 mg, 84%, *d.r.* = 98:2) as a colourless oil. Here the stereochemical identity for a carbon bearing the hydroxyl group shown with squiggly line is unspecified. This might be due to the possible attack of the lithiated intermediate on faces of benzadehyde. Secondly, in the ^1^H-NMR spectrum, the proton coupling at this position is not clear hence it is given as a multiplet. Spectral data matched those reported in the literature [3]. *R*_f_ = 0.40 (10% EtOAc in petroleum ether); IRν_max_ (neat)/cm^−1^ 3447, 2970, 1680, 1442, 1314, 1070; ^1^H-NMR (CDCl_3_, 270 MHz); δ: 7.30–7.19 (5 H, m, arom. H), 5.93–5.88 (1 H, m, C*H*(OH)Ph), 4.42–4.30 (1 H, m, C*H*OCb), 4.21 (2 H, d, *J* = 5.7 Hz, C*H*_2_OC), 3.98–3.61 (2 H, m, 2 × C*H*), 3.58–3.51 (1 H, m, C*H*), 3.47 (1 H, br s, O*H*), 1.92 (2 H, dd, *J* = 8.9, 4.8 Hz, C*H*_2_), 1.37 (3 H, s, C*H*_3_), 1.31 (3 H, s, C*H*_3_), 1.16 (12 H, d, *J* = 6.9 Hz, 4 × C*H*_3_); ^13^C-NMR (CDCl_3_, 100 MHz); δ: 155.5 (*C*=O), 133.2 (*C*-Ar), 130.1 (2 × *C*H-Ar), 129.8 (2 × *C*H-Ar), 128.5 (*C*H-Ar), 119.2 (*C*(CH_3_)_2_), 73.6 (*C*HOH), 66.6 (*C*HOCb), 64.6 (*C*HCH_2_O), 60.9 (*C*H_2_O), 45.6 (2 × CHN), 33.3 (*C*H_2_), 25.7 (*C*H_3_), 21.3 (*C*H_3_), 20.6 (4 × CH_3_); HRMS (ESI) calcd. for C_21_H_33_NO_5_Na (M+Na): 389.2359; Found: 389.2364; ${\left[\text{α}\right]}_{D}^{23}$ −17.2 (conc. 1.1, CH_3_OH); Anal. Calcd for C_21_H_33_NO_5_: C, 66.46; H, 8.76; N, 3.69; Found: C, 66.50; H, 8.72; N, 3.70.

### *3.6.* (R)-3-((S)-2,2-Dimethyl-1,3-dioxolan-4-yl)-1-hydroxy-1,1-diphenylpropan-2-yldiisopropyl-carbamate *(**4a-III**)*

To a solution of carbamate (0.208 mg, 0.75 mmol) in Et_2_O (5 mL) at –78 °C was added *s*BuLi (1.3 M in cyclohexane, 0.84 mL, 1.05 mmol,) drop-wise. This mixture was then stirred at –78 °C for 5 h followed by addition of benzophenone (1.75 mL, 2.00 mmol). The reaction was stirred for 4h and then warmed to r.t. and stirred for 12 h. The reaction mixture was then cooled to 0 °C and a solution of 2 N HCl (10 mL) was added drop-wise. The layers were separated and the aqueous layer was extracted with Et_2_O (3 × 15 mL). The combined organic layers were dried over MgSO_4_ and concentrated *in vacuo*. The crude product with 4 diastereomers was purified by flash chromatography (SiO_2_, 10% EtOAc/petroleum ether) to give the major diastereomeric product **4a-III** (170 mg, 84%, *d.r.* = 98:2) as a colourless oil. Spectral data matched those reported in the literature [3]. *R*_f_ = 0.35 (10% EtOAc in petroleum ether); IRν_max_ (neat)/cm^−1^ 3432, 2890, 1690, 1438, 1060; ^1^H-NMR (CDCl_3_, 270 MHz); δ: 7.28–7.09 (10 H, m, arom. H), 4.35–4.31 (1 H, m, C*H*OCb), 4.11 (2 H, d, *J* = 5.4 Hz, C*H*_2_OC), 3.96–3.70 (2 H, m, C*H*), 3.52–3.46 (1 H, m, C*H*), 2.90 (1 H, br s, O*H*), 1.91 (2 H, dd, *J* = 8.6, 4.9 Hz, C*H*_2_), 1.35 (3 H, s, C*H*_3_), 1.29 (3 H, s, C*H*_3_), 1.14 (12 H, d, *J* = 6.9 Hz, 4 × C*H*_3_); ^13^C-NMR (CDCl_3_, 100 MHz); δ: 153.0 (*C*=O), 145.2 (2 × C-Ar), 129.8 (4 × CH-Ar), 128.5 (4 × CH-Ar), 119.2 (*C*(CH_3_)_2_), 83.6 (*C*-OH), 82.0 (*C*HOCb), 71.6 (*C*HCH_2_O), 60.9 (*C*H_2_O), 45.6 (2 × CHN), 33.3 (*C*H_2_), 25.7 (*C*H_3_), 21.3 (*C*H_3_), 20.6 (4 × CH_3_); HRMS (ESI) calcd. for C_27_H_37_NO_5_Na (M+Na): 458.2031; Found: 458.2060; ${\left[\text{α}\right]}_{D}^{23}$ +27.3 (conc.1.5, CH_3_OH).

### 3.7. (S)-2-((S)-2,2-Dimethyl-1,3-dioxolan-4-yl)-1-(trimethylstannyl)ethyl Diisopropylcarbamate *(**4a-II**)*

To a solution of carbamate (0.208 mg, 0.75 mmol) in Et_2_O (5 mL) at –78 °C was added *s*BuLi (1.3 M in cyclohexane/hexane, 16.2 mL, 22.0 mmol) drop-wise at –78 °C followed by trapping with trimethyltin chloride (1 M in hexane, 0.58 mL, 1.7 mmol). The crude material with 4 diastereomers was purified by flash column chromatography (SiO_2_, EtOAc/petroleum ether, 1:4) to give the major diastereomer **4a-II** as a colourless oil (122mg, 78%, *d.r.* = 98:2). Spectral data matched those reported in the literature [3]. *R*_f_ = 0.43 (10% EtOAc in petrol); ^1^H-NMR (CDCl_3_, 270 MHz); δ: 4.38 (1 H, ddd, *J* = 11.3, 9.2, 5.2 Hz, C*H*S*_n_*), 4.10 (2 H, d, *J* = 5.5 Hz, C*H*_2_OC(CH_3_)_2_), 3.70–3.60 (1 H, m, C*H*CH_2_O), 3.51–3.46 (2 H, m, 2 × C*H*), 1.90 (2 H, dd, *J* = 8.2, 5.0 Hz, C*H*_2_), 1.28 (3 H, s, C*H*_3_), 1.12 (3 H, s, C*H*_3_), 1.15 (12 H, d, *J* = 6.9 Hz, 4 × C*H*_3_), 0.69 (9 H, s, Sn(C*H*_3_)_3_); ^13^C-NMR (CDCl_3_, 67.5 MHz); δ: 153.2 (*C*=O), 119.2 (*C*(CH_3_)_2_), 77.6 (*CH*CH_2_O), 68.9 (*C*H_2_O), 63.0 (*C*HS*_n_*), 55.5 (2 × *C*HN), 32.4 (*C*H_2_), 25.8 (2 × *C*H_3_), 21.4 (3 × *C*H_3_), 21.2 (4 × *C*H_3_); HRMS (ESI) calcd. for C_17_H_35_NO_4_SnNa (M+Na): 437.1588; Found: 437.1590; ${\left[\text{α}\right]}_{D}^{23}$ +11.3 (conc. 1.1, CH_3_OH); Anal. Calcd for C_17_H_35_NO_4_Sn: C, 46.81; H, 8.09; N, 3.21; Found: C, 46.80; H, 8.10; N, 3.17.

### 3.8. (1R,2R,Z)-5-((R)-2,2-Dimethyl-1,3-dioxalan-4-yl)-2-methyl-1-phenylpent-3-en-1-ol *(**8-I**)*

*s*BuLi (1.3 M in cyclohexane, 0.84 mL, 1.05 mmol) was added at –78 °C to a solution of acetonide carbamate (208 mg, 0.75 mmol). After stirring for 5 h, *B*-vinyl,Me-9-BBN (1 M in Et_2_O, 2.0 mL, 1.12 mmol) was added at −78 °C, followed by trapping with benzaldehyde (2.0 mL, 2.0 mmol) at −78 °C. The crude product was purified by flash column chromatography (SiO_2_, petroleum ether/EtOAc 10:2) followed by removal of residual benzaldehyde at reduced pressure, to give the *alcohol* (40 mg, 56%, *d.r.* = 90:10) as a colourless oil. *R*_f_ = 0.34 (20% EtOAc in petrol); ${\left[\text{α}\right]}_{D}^{24}$ +10.4 (conc. 0.50, CH_2_Cl_2_); ^1^H-NMR (400 MHz; CDCl_3_); δ: 7.37–7.28 (5 H, m, arom.H), 5.64 (1 H, ddd, *J* = 10.9, 6.1, 6.1 Hz, (acetonide)CH_2_C*H*=CH), 5.52 (1 H, dddd, *J* = 10.9, 7.5, 1.4, 1.4 Hz, (acetonide)CH_2_CH=C*H*), 4.29 (1 H, dd, *J* = 8.5, 2.9 Hz, C*H*OH), 4.17–4.12 (1 H, m, CHC*H*HOC(CH_3_)_2_), 4.09 (1 H, ddd, *J* = 7.0, 2.3, 0.49 Hz, CHCH*H*OC(CH_3_)_2_), 3.58 (1 H, dddd, *J* = 7.0, 7.0, 6.2, 6.2 Hz, C*H*CH_2_OC(CH_3_)_2_), 2.90 (1 H, d, *J* = 2.9 Hz, OH), 2.79 (1 H, ddq, *J* = 8.5, 7.5, 6.7 Hz, C*H*CH_3_), 2.48 (1 H, dddd, *J* = 7.0, 6.1, 4.9, 1.4 Hz, C*H*HCH=CH), 2.30 (1 H, dddd, *J* = 7.1, 6.1, 4.9, 1.4 Hz, CH*H*CH=CH), 1.50 (3 H, d, *J* = 0.49 Hz, C(C*H*_3_CH_3_)), 1.38 (3 H, d, *J* = 0.50 Hz, C(CH_3_CH_3_)), 0.79 (3 H, t, *J* = 6.7 Hz, CH_3_); ^13^C-NMR (100.5 MHz; CDCl_3_); δ: 143.0 (*C*-Ar), 135.5 (*C*=C), 128.4 (2 × *C*H-Ar), 128.3 (*C*H-Ar), 127.7 (2 × *C*H-Ar), 127.1 (C=*C*), 126.9 (*C*-(CH_3_)_2_), 78.9 (*C*HOH), 75.6 (*C*H(O)C(CH_3_)_2_), 69.3 (*C*H_2_), 40.6 (*C*H), 32.0 (*C*H_2_), 27.0 (*C*H_3_), 25.7 (*C*H_3_), 17.7 (*C*H_3_); IRν_max_ (neat/cm^−1^): 3421, 2929, 1691, 1457, 1036, 700;MS(ESI) *m*/*z* (%) 299 (29.1, M+Na), 259 (12.7, M–OH), 183 (4.4), 168 (5.2); HRMS (ESI) calcd. for C_17_H_24_O_3_Na (M+Na): 299.1621; Found: 299.1617; EA Anal. Calcd. for C_17_H_24_O_3_: C, 73.88; H, 8.75; Found: C, 73.85; H, 8.74. ^1^H-NMR signals for the minor product (400 MHz; CDCl_3_); δ: 7.37–7.28 (5 H, m, arom.H), 5.65–5.64 (1 H, m, (acetonide)CH_2_C*H*=CH), 5.61–5.60 (1 H, m, (acetonide)CH_2_CH=C*H*), 4.31 (1 H, dd, *J* = 1.9, 8.0 Hz, C*H*OH), 4.18–4.12 (1 H, m, CHC*H*HOC(CH_3_)_2_), 4.09–4.07 (1 H, m, CHCH*H*OC(CH_3_)_2_), 3.61–3.57 (1 H, m, C*H*CH_2_OC(CH_3_)_2_), 2.90 (1 H, d, *J* = 1.9 Hz, OH), 2.79–2.61 (1 H, m, C*H*CH_3_), 2.53–2.51(1 H, m, C*H*HCH=CH), 2.43–2.41 (1 H, m, CH*H*CH=CH), 1.50–148 (3 H, C(C*H*_3_CH_3_)), 1.39–137 (3 H, m, C(CH_3_CH_3_)), 0.65 (3 H, d, *J* = 6.2 Hz, CH_3_)

### 3.9. (1R,2R,Z)-1-Cyclohexyl-5-((R)-2,2-dimethyl-1,3-dioxalan-4-yl)-2-methylpent-3-en-1-ol *(**8-II**)*.

*s*BuLi (1.3 M in cyclohexane, 0.84 mL, 1.05 mmol) was added at –78 °C to a solution of acetonide carbamate (208 mg, 0.75 mmol). After stirring for 5 h, *B*-vinyl,Me-9-BBN (1 M in Et_2_O, 2.0 mL, 1.12 mmol) was added at −78 °C, followed by trapping with cyclohexanecarboxaldehyde (2.0 mL, 2.0 mmol) at –78 °C. The crude product was purified by flash column chromatography (SiO_2_, petroleum ether/EtOAc 10:2) followed by removal of residual aldehyde at reduced pressure, to give the *alcohol* (48 mg, 54%, *d.r.* = 95:5) as a colourless oil. *R*_f_ = 0.38 (20% EtOAc in petrol); ${\left[\text{α}\right]}_{D}^{24}$ −19.5 (conc. 0.49, CH_2_Cl_2_); ^1^H-NMR (400 MHz; C_6_D_6_); δ: 5.44–5.35 (2H, m, (acetonide)CH_2_C*H*=C*H*CH(CH_3_), 3.85 (1 H, dddd, *J* = 7.7, 7.7, 5.9, 4.7 Hz, CHC*H*HOC(CH_3_)_2_), 3.70 (1 H, dd, *J* = 7.7, 5.9 Hz, CHCH*H*OC(CH_3_)_2_), 3.34 (1 H, dd, *J* = 7.7, 7.7 Hz, C*H*OH), 3.01 (1 H, dddd, *J* = 7.6, 7.6, 3.7, 3.7 Hz, C*H*CH_2_OC(CH_3_)_2_), 2.65–2.55 (1 H, m, C*H*CH_3_), 2.33–2.21 (1 H, m, CH of cyclohexane), 2.00–1.93 (2 H, m, C*H*_2_CH=CH), 1.79–1.51 (6 H, m, 3 × C*H*_2_), 1.47 (3 H, br s, C(C*H*_3_CH_3_)), 1.42–1.36 (2 H, m, CH_2_), 1.30 (3 H, d, *J* = 0.48 Hz, C(CH_3_C*H*_3_)), 1.28–1.16 (4 H, m, 2 × C*H*_2_), 0.86 (3 H, d, *J* = 6.7 Hz, C*H*_3_); ^13^C-NMR (100.5 MHz; C_6_D_6_); δ: 135.2 (*C*=C), 125.8 (C=*C*), 108.9 (*C*(CH_3_)_2_), 78.7 (*C*H), 75.4 (*C*H), 69.1 (*C*H_2_), 40.3 (*C*H), 35.1 (*C*H), 32.0 (*C*H_2_), 30.7 (*C*H_2_), 26.9 (*C*H_2_), 26.8 (2 × CH_2_), 26.5 (*C*H_2_), 26.2 (*C*H_3_), 25.6 (*C*H_3_), 17.4 (*C*H_3_); IR ν_max_ (neat/cm^−1^): 3431, 1608, 1496, 1452, 1029, 962; MS(ESI) *m*/*z *(%) 305 (100, M+Na), 296 (21.3), 207 (8.2), 152 (7.0), 101 (6.9); HRMS (ESI) calcd. for C_17_H_30_O_3_Na (M+Na): 305.2089; Found: 305.2087; EA Anal. Calcd. for C_17_H_30_O_3_: C, 72.30; H, 10.71; Found: C, 72.63; H, 10.46; ); ^1^H-NMR signals for the minor product (400 MHz; C_6_D_6_); δ: 5.48–5.44 (2H, m, (acetonide)CH_2_C*H*=C*H*CH(CH_3_), 3.87–3.85 (1 H, m, CHC*H*HOC(CH_3_)_2_), 3.70 (1 H, dd, *J* = 7.4, 5.9 Hz, CHCH*H*OC(CH_3_)_2_), 3.34 (1 H, dd, *J* = 5.7, 7.9 Hz, C*H*OH), 3.03–3.02 (1 H, m, C*H*CH_2_OC(CH_3_)_2_), 2.69–2.66 (1 H, m, C*H*CH_3_), 2.35–2.33 (1 H, m, CH of cyclohexane), 2.02–2.00 (2 H, m, C*H*_2_CH=CH), 1.85–1.80 (6 H, m, 3 × C*H*_2_), 1.48 (3 H, br s, C(C*H*_3_CH_3_)), 1.42–1.36 (2 H, m, CH_2_), 1.31 (3 H, s, C(CH_3_C*H*_3_)), 1.36–1.33 (4 H, m, 2 × C*H*_2_), 0.88 (3 H, d, *J* = 6.8 Hz, C*H*_3_).

### 3.10. (4R,5S,Z)-1-((R)-2,2-Dimethyl-1,3-dioxolan-4-yl)-4-methylnon-2-en-5-ol *(**8-III**)*

*s*BuLi (1.3 M in cyclohexane, 0.80 mL, 1.00 mmol) was added at –78 °C to a solution of acetonide carbamate (200 mg, 0.70 mmol). After stirring for 5 h, *B*-vinyl,Me-9-BBN (1 M in Et_2_O, 1.6 mL, 1.00 mmol) was added at −78 °C, followed by trapping with *n*-butyraldehyde (2.0 mL, 2.0 mmol) at −78 °C. The crude product was purified by flash column chromatography (SiO_2_, petroleum ether/EtOAc 10:2) followed by removal of residual aldehyde at reduced pressure, to give the alcohol (57 mg, 60%, *d.r.* = 92:8) as a colourless oil. *R*_f_ = 0.36 (20% EtOAc in petroleum ether); ${\left[\text{α}\right]}_{D}^{24}$ +21.9 (conc. 0.50, CH_2_Cl_2_); ^1^H-NMR (400 MHz; CDCl_3_); δ: 5.54 (1 H, ddd, *J* = 10.5, 7.1, 7.1 Hz, (acetonide)CH_2_C*H*=CH), 5.52 (1 H, dddd, *J* = 10.4, 7.4, 1.3, 1.3 Hz, (acetonide)CH_2_CH=C*H*), 4.27 (1 H, dd, *J* = 8.3, 2.5 Hz, C*H*OH), 4.15–4.11 (1 H, m, CHC*H*HOC(CH_3_)_2_), 4.05 (1 H, ddd, *J* = 6.8, 2.5, 0.56 Hz, CHCH*H*OC(CH_3_)_2_), 3.58 (1 H, dddd, *J* = 6.9, 6.9, 6.1, 6.1 Hz, C*H*CH_2_OC(CH_3_)_2_), 2.90 (1 H, d, *J* = 2.8 Hz, OH), 2.79–2.74 (1 H, m, C*H*CH_3_), 2.48 (1 H, dddd, *J* = 7.0, 6.1, 4.9, 1.4 Hz, C*H*HCH=CH), 2.30 (1 H, dddd, *J* = 7.1, 6.1, 4.9, 1.4 Hz, CH*H*CH=CH), 1.59 (3 H, d, *J* = 0.45 Hz, C(C*H*_3_CH_3_)), 1.57–1.50 (6 H, m, CH_3_(C*H*_2_)_3_), 1.42 (6 H, s, C(CH_3_CH_3_)), 0.70 (6 H, m, CHC*H*_3_and C*H*_3_(CH_2_)_2_CHOH); ^13^C-NMR (100.5 MHz; CDCl_3_); δ: 135.0 (*C*=C), 127.1 (C=*C*), 126.9 (*C*-(CH_3_)_2_), 78.9 (*C*HOH), 75.6 (*C*H(O)C(CH_3_)_2_), 69.3 (*C*H_2_), 40.6 (*C*H), 32.0 (3 × CH_2_), 27.0 (*C*H_3_), 25.7 (*C*H_3_), 17.7 (2 × CH_3_); IR ν_max_ (neat/cm^−1^): 3422, 2925, 1700, 1460, 1039, 709; MS(ESI) *m*/*z* (%) 299 (29.1, M+Na), 259 (12.7, M–OH), 183 (4.4), 168 (5.2); HRMS (ESI) calcd. for C_15_H_28_O_3_Na (M+Na): 279.2139; Found: 299.2100; EA Anal. Calcd. for C_15_H_28_O_3_: C, 72.86; H, 8.73; Found: C, 72.85; H, 8.69. ^1^H-NMR signals for the minor product (400 MHz; CDCl_3_); δ: 5.56 (1 H, ddd, *J* = 13.3, 5.6, 5.7 Hz, (acetonide)CH_2_C*H*=CH), 5.57–5.54 (1 H, m, (acetonide)CH_2_CH=C*H*), 4.30–4.26 (1 H, m, C*H*OH), 4.16–4.15 (1 H, m, CHC*H*HOC(CH_3_)_2_), 4.03 (1 H, ddd, *J* = 6.3, 1.9, 0.80 Hz, CHCH*H*OC(CH_3_)_2_), 3.58 (1 H, dddd, *J* = 7.1, 7.1, 5.6, 5.6 Hz, C*H*CH_2_OC(CH_3_)_2_), 2.91–2.90 (1 H, m, OH), 2.83–2.80 (1 H, m, C*H*CH_3_), 2.48 (1 H, dddd, *J* = 5.8, 5.6, 5.4, 1.8 Hz, C*H*HCH=CH), 2.32–2.31 (1 H, m, CH*H*CH=CH), 1.60 (3 H, s, C(C*H*_3_CH_3_)), 1.59–1.56 (6 H, m, CH_3_(C*H*_2_)_3_), 1.44 (6 H, s, C(CH_3_CH_3_)), 0.74–0.70 (6 H, m, CHC*H*_3_ and C*H*_3_(CH_2_)_2_CHOH).

### 3.11. (1R,2R)-2-((Z)-3-((R)-2,2-Dimethyl-1,3-dioxolan-4-yl)prop-1-en-1-yl)-1-phenylhexan-1-ol *(**8-IV**)*.

*s*BuLi (1.3 M in cyclohexane, 0.80 mL, 1.00 mmol) was added at –78 °C to a solution of acetonide carbamate (200 mg, 0.70 mmol). After stirring for 5 h, *B*-vinyl,Bu-9-BBN (1 M in Et_2_O, 1.4 mL, 1.00 mmol) was added at −78 °C, followed by trapping with benzaldehyde (2.0 mL, 2.0 mmol) at –78 °C. The crude product was purified by flash column chromatography (SiO_2_, petroleum ether ether/EtOAc 10:2) followed by removal of residual aldehyde at reduced pressure, to give the alcohol (44 mg, 48%, *d.r.* = 90:10) as a colourless oil. *R*_f_ = 0.34 (20% EtOAc in petroleum ether); ${\left[\text{α}\right]}_{D}^{25}$ ‒14.5 (conc. 0.50, CH_2_Cl_2_) ^1^H-NMR (400 MHz; CDCl_3_); δ: 7.37–7.28 (5 H, m, arom.H), 5.63 (1 H, ddd, *J* = 10.9, 6.0, 6.0 Hz, (acetonide)CH_2_C*H*=CH), 5.50 (1 H, dddd, *J* = 10.7, 7.0, 1.2, 1.2 Hz, (acetonide)CH_2_CH=C*H*), 4.32 (1 H, dd, *J* = 8.5, 3.9 Hz, C*H*OH), 4.17–4.12 (1 H, m, CHC*H*HOC(CH_3_)_2_), 4.09 (1 H, ddd, *J* = 7.0, 2.3, 0.49 Hz, CHCH*H*OC(CH_3_)_2_), 3.58 (1 H, dddd, *J* = 7.0, 7.0, 6.2, 6.2 Hz, C*H*CH_2_OC(CH_3_)_2_), 2.90 (1 H, d, *J* = 2.9 Hz, OH), 2.62 (1 H, ddq, *J* = 8.5, 7.5, 6.7 Hz, C*H*CH_3_), 2.48 (1 H, dddd, *J* = 7.0, 6.1, 4.9, 1.4 Hz, C*H*HCH=CH), 2.30 (1 H, dddd, *J* = 7.1, 6.1, 4.9, 1.4 Hz, CH*H*CH=CH), 1.87–180 (4H, m), 1.50 (2 H, t, *J* = 0.49 Hz, C(C*H*_2_CH_3_)), 1.38 (3 H, d, *J* = 0.50 Hz, C(CH_3_CH_3_)), 0.79 (3 H, t, *J* = 6.7 Hz, CH_3_); ^13^C-NMR (100.5 MHz; CDCl_3_); δ: 142.0 (*C*-Ar), 133.5 (*C*=C), 129.4 (2 × CH-Ar), 128.3 (*C*H-Ar), 127.7 (2 × CH-Ar), 127.4 (C=*C*), 126.9 (*C*-(CH_3_)_2_), 79.9 (*C*HOH), 75.6 (*C*H(O)C(CH_3_)_2_), 69.3 (4 × CH_2_), 40.6 (*C*H), 32.0 (4 × CH_2_), 27.0 (*C*H_3_), 25.7 (*C*H_3_), 17.7 (*C*H_3_); IR ν_max_ (neat/cm^−1^): 3400, 2910, 1695, 1454, 1037, 709; HRMS (ESI) calcd. for C_20_H_30_O_3_Na (M+Na): 341.1432; Found: 341.1480; EA Anal. Calcd. for C_20_H_30_O_3_: C, 71.88; H, 8.75; Found: C, 71.87; H, 8.74. ^1^H-NMR signals for the minor product (400 MHz; CDCl_3_); δ: 7.40–7.33 (5 H, m, arom.H), 5.65 (1 H, ddd, *J* = 13.9, 5.2, 5.2 Hz, (acetonide)CH_2_C*H*=CH), 5.53 (1 H, ddd, *J* = 13.8, 7.0, 3.2 Hz, (acetonide)CH_2_CH=C*H*), 4.34–4.32 (1 H, m, C*H*OH), 4.21–4.19 (1 H, m, CHC*H*HOC(CH_3_)_2_), 4.09 (1 H, ddd, *J* = 7.0, 2.3, 0.49 Hz, CHCH*H*OC(CH_3_)_2_), 3.58 (1 H, dddd, *J* = 7.1, 7.1, 6.1, 6.0 Hz, C*H*CH_2_OC(CH_3_)_2_), 2.90 (1 H, d, *J* = 2.9 Hz, OH), 2.66–2.63 (1 H, m, C*H*CH_3_), 2.48 (1 H, dddd, *J* = 7.0, 5.2, 5.2, 1.5 Hz, C*H*HCH=CH), 2.30 (1 H, ddd, *J* = 7.1, 5.2, 5.2, 1.6 Hz, CH*H*CH=CH), 1.89–1.83 (4H, m), 1.55–1.52 (2 H, m, *J* = 0.51 Hz, C(C*H*_2_CH_3_)), 1.38 (3 H, s, C(CH_3_CH_3_)), 0.81 (3 H, t, *J* = 6.5 Hz, CH_3_)

### 3.12. (1S,2R)-1-Cyclohexyl-2-((Z)-3-((R)-2,2-dimethyl-1,3-dioxolan-4-yl)prop-1-en-1-yl)hexan-1-ol *(**8-V**)*

*s*BuLi (1.3 M in cyclohexane, 0.80 mL, 1.00 mmol) was added at –78 °C to a solution of acetonide carbamate (200 mg, 0.70 mmol) . After stirring for 5 h, *B*-vinyl,Bu-9-BBN (1 M in Et_2_O, 1.6 mL, 1.00 mmol) was added at −78 °C, followed by trapping with cyclohexanecarboxaldehyde (2.2 mL, 2.0 mmol) at –78 °C. The crude product was purified by flash column chromatography (SiO_2_, petroleum ether/EtOAc 10:2) followed by removal of residual aldehyde at reduced pressure, to give the alcohol (42 mg, 52%, *d.r.* = 94:6) as a colourless oil. *R*_f_ = 0.38(20% EtOAc in petroleum ether); ${\left[\text{α}\right]}_{D}^{25}$ −18.4 (conc. 0.49, CH_2_Cl_2_); ^1^H-NMR (400 MHz; C_6_D_6_); δ: 5.60–5.45 (2 H, m, (acetonide)CH_2_C*H*=C*H*CH(CH_3_), 4.01 (1 H, dddd, *J* = 7.7, 7.7, 5.9, 4.7 Hz,CHC*H*HOC(CH_3_)_2_), 3.70 (1 H, dd, *J* = 7.7, 5.9 Hz, CHCH*H*OC(CH_3_)_2_), 3.33 (1 H, dd, *J* = 7.7, 7.7 Hz, C*H*OH), 3.00 (1 H, dddd, *J* = 7.6, 7.6, 3.7, 3.7 Hz,C*H*CH_2_OC(CH_3_)_2_), 2.75–2.55 (1 H, m, C*H*CH_3_), 2.38–2.26 (1 H, m, CH of cyclohexane), 2.10–1.98 (2 H, m, C*H*_2_CH=CH), 1.79–1.51 (6 H, m, 3 × C*H*_2_), 1.45 (3 H, br s, C(C*H*_3_CH_3_)), 1.42–1.34 (4 H, m, CH_2_), 1.30 (3 H, d, *J* = 0.48 Hz, C(CH_3_C*H*_3_)), 1.28–1.13 (6 H, m, 2 × C*H*_2_), 0.86 (3 H, d, *J* = 6.7 Hz, C*H*_3_); ^13^C-NMR (100.5 MHz; C_6_D_6_);;δ: 136.2 (*C*=C), 126.8 (C=*C*), 109.9 (*C*(CH_3_)_2_), 79.7 (*C*H), 75.4 (*C*H), 69.1 (*C*H_2_), 40.3 (*C*H), 35.1 (*C*H), 32.0 (*C*H_2_), 30.7 (*C*H_2_), 26.9 (*C*H_2_), 26.8 (2 × CH_2_), 26.5 (*C*H_2_), 26.2 (*C*H_3_), 25.6 (*C*H_3_), 17.4 (*C*H_3_); IR ν_max_ (neat/cm^−1^): 3441, 1608, 1496, 1470, 1029, 962; HRMS (ESI) calcd. for C_20_H_36_O_3_Na (M+Na): 347.2065; Found: 347.2060; EA Anal. Calcd. for C_20_H_36_O_3_: C, 73.31; H, 10.01; Found: C, 71.33; H, 10.06. ^1^H-NMR signals for the minor product (400 MHz; C_6_D_6_); δ: 5.66–5.61 (2 H, m, (acetonide)CH_2_C*H*=C*H*CH(CH_3_), 4.07–4.04 (1 H, m, CHC*H*HOC(CH_3_)_2_), 3.74 (1 H, dd, *J* = 7.4, 5.0 Hz, CHCH*H*OC(CH_3_)_2_), 3.36–3.34 (1 H, m, C*H*OH), 3.02 (1 H, dddd, *J* = 7.2, 7.2, 4.1, 3.7 Hz,C*H*CH_2_OC(CH_3_)_2_), 2.75–2.55 (1 H, m, C*H*CH_3_), 2.46–2.39 (8 H,m, CH of cyclohexane), 2.11–1.98 (2 H, m, C*H*_2_CH=CH), 1.47 (3 H, br s, C(C*H*_3_CH_3_)), 1.42–1.34 (4 H, m, CH_2_), 1.30 (3 H, d, *J* = 0.48 Hz, C(CH_3_C*H*_3_)), 1.30–1.28 (2 H, m, 2 × C*H*_2_), 0.86 (3 H, t, *J* = 6.2 Hz, C*H*_3_).

## 4. Conclusions

The lithiation of 2-(2,2-dimethyl-1,3-dioxolan-4-yl)ethyl diisopropylcarbamate (**1**) can be achieved with extreme selectivity by the selection of appropriate chelating agents. Furthermore, this application of chelation-directed asymmetric lithiation and subsequent borylation and allylation reactions then gave (*Z*)-*anti-*homoallylic alcohols **8**(**I**–**V**) in good yield and excellent *d.r*. with full stereocontrol.

## Supplementary Materials

Supplementary materials can be accessed at: http://www.mdpi.com/1420-3049/20/06/9890/s1.
