# Supplementary material for: Chelation-Assisted Substrate-Controlled Asymmetric Lithiation-Allylboration of Chiral Carbamate 1,2,4-Butanetriol Acetonide
Source: Molecules. 2015 May 28;20(6):9890–905. doi: 10.3390/molecules20069890 (PMC6272734; doi:10.3390/molecules20069890)
Supplement: Supplementary file 1 [file molecules-20-09890-s001.pdf]

Supplementary Materials

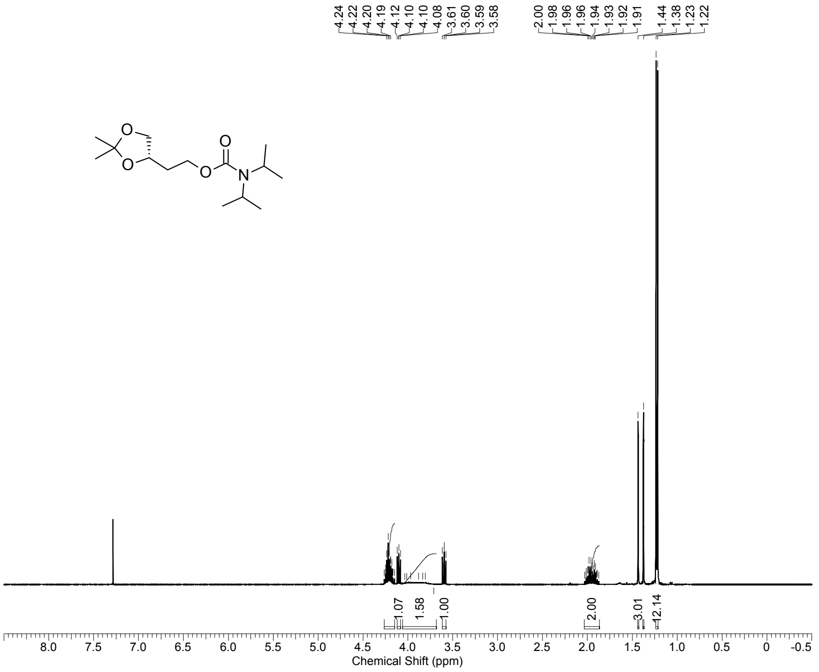

Figure S1. ^{1}H-NMR Spectrum for (*S*)-2-(2,2-dimethyl-1,3-dioxolan-4-yl)ethyl diisopropylcarbamate.

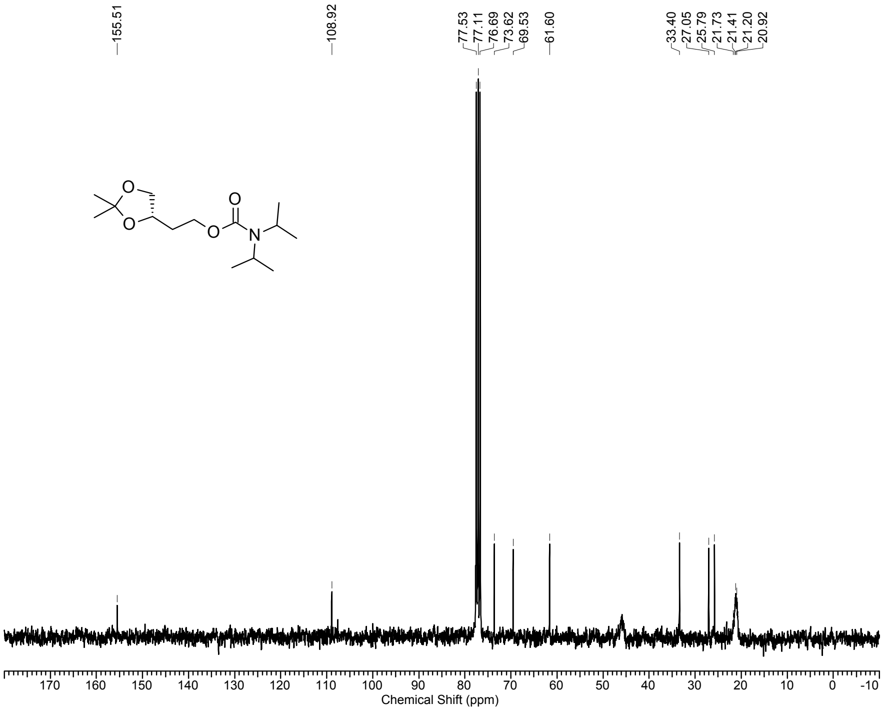

Figure S2. ^{13}C-NMR Spectrum for (*S*)-2-(2,2-dimethyl-1,3-dioxolan-4-yl)ethyl diisopropylcarbamate.

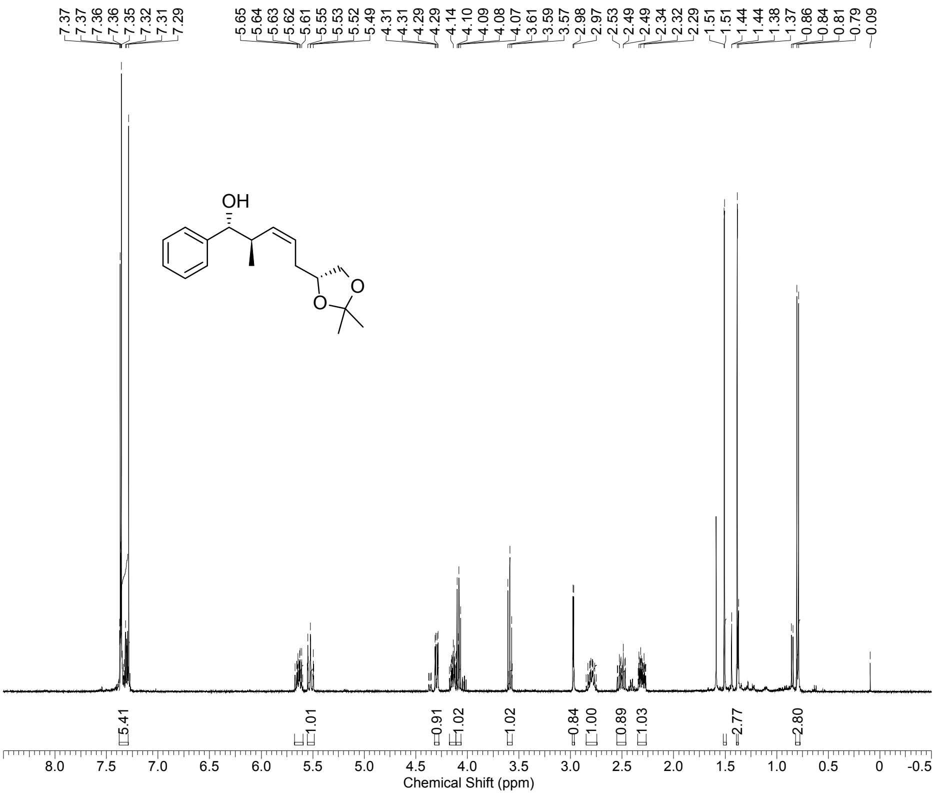

Figure S3. ^{1}H-NMR Spectrum for (1*R*,2*R*,*Z*)-5-((*R*)-2,2-dimethyl-1,3-dioxalan-4-yl)-2-methyl-1-phenylpent-3-en-1-ol (**8-I**).

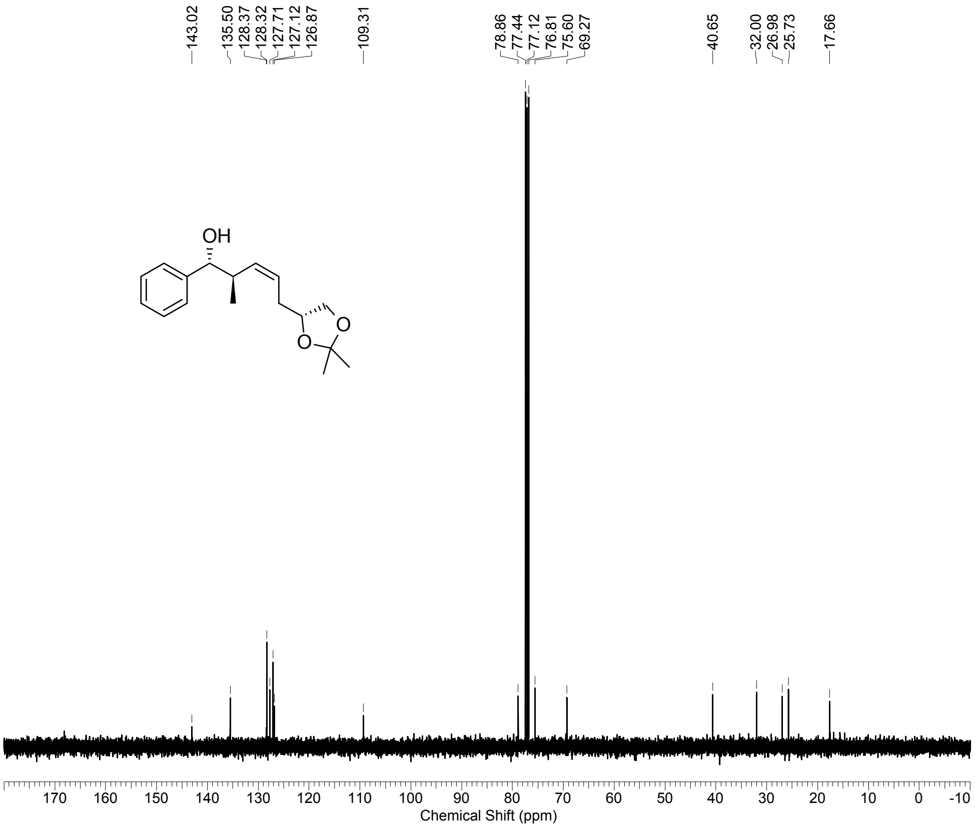

Figure S4. ^{13}C-NMR Spectrum for (1*R*,2*R*,*Z*)-5-((*R*)-2,2-dimethyl-1,3-dioxalan-4-yl)-2-methyl-1-phenylpent-3-en-1-ol (**8-I**).

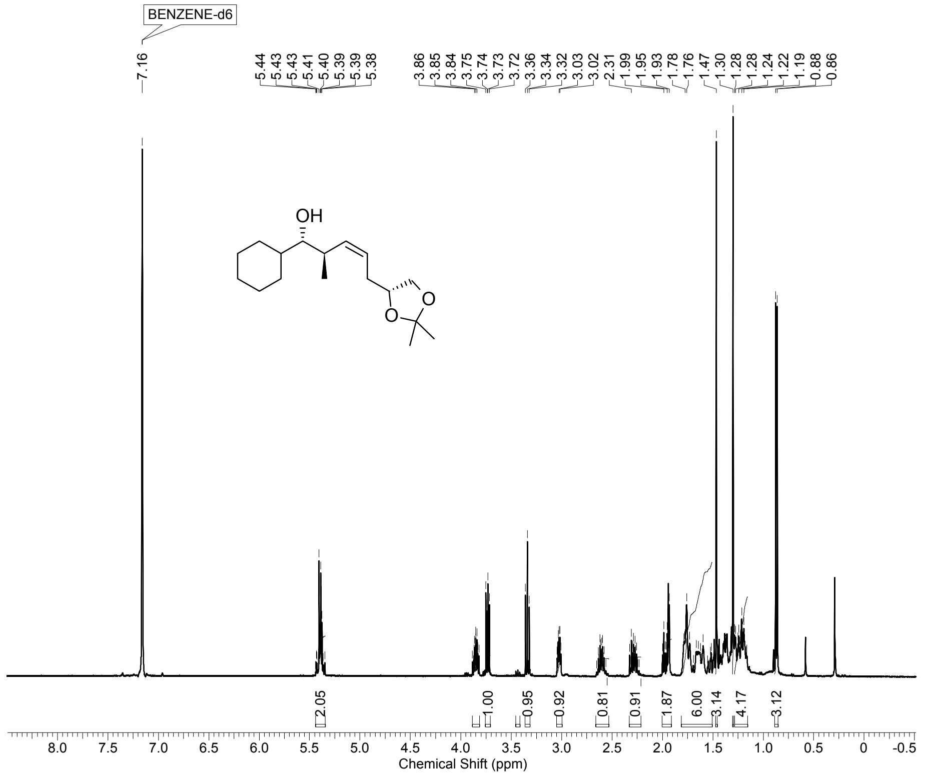

Figure S5. ^{1}H-NMR Spectrum for (1*R*,2*R*,*Z*)-1-cyclohexyl-5-((*R*)-2,2-dimethyl-1,3-dioxalan-4-yl)-2-methylpent-3-en-1-ol (**8-II**).

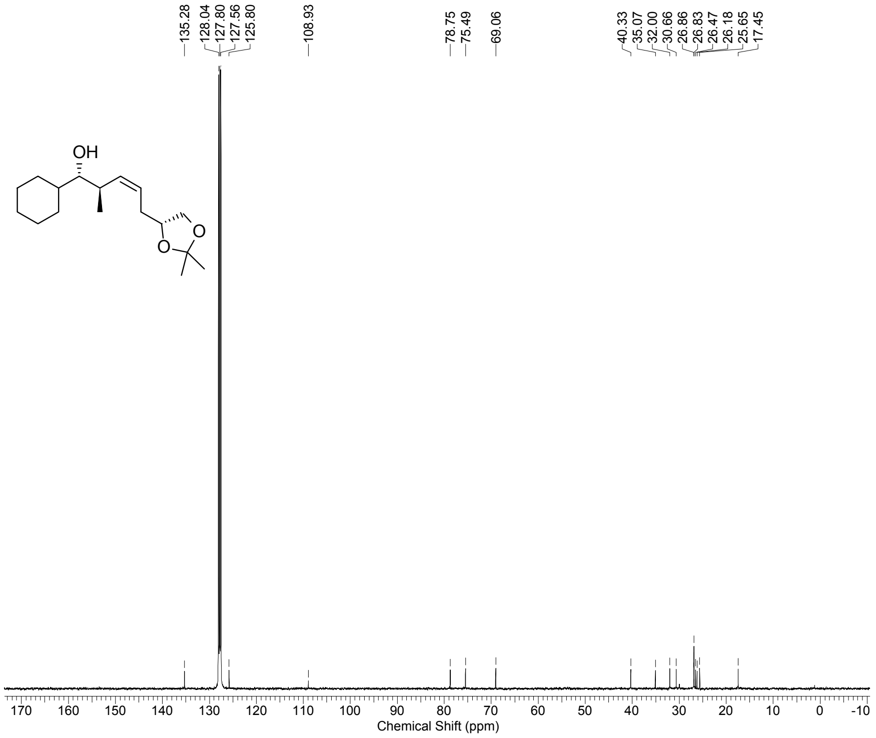

Figure S6. ^{13}C-NMR Spectrum for (1*R*,2*R*,*Z*)-1-cyclohexyl-5-((*R*)-2,2-dimethyl-1,3-dioxalan-4-yl)-2-methylpent-3-en-1-ol (**8-II**).
